# Comparison of Summer and Winter Objectively Measured Physical Activity and Sedentary Behavior in Older Adults: Age, Gene/Environment Susceptibility Reykjavik Study

**DOI:** 10.3390/ijerph14101268

**Published:** 2017-10-21

**Authors:** Nanna Yr Arnardottir, Nina Dora Oskarsdottir, Robert J. Brychta, Annemarie Koster, Dane R. Van Domelen, Paolo Caserotti, Gudny Eiriksdottir, Johanna E. Sverrisdottir, Erlingur Johannsson, Lenore J. Launer, Vilmundur Gudnason, Tamara B. Harris, Kong Y. Chen, Thorarinn Sveinsson

**Affiliations:** 1 Faculty of Education, University of Akureyri, Nordurslod 2, 600 Akureyri, Iceland; 2 Icelandic Heart Association, Holtasmári 1, 201 Kópavogur, Iceland; ndo2@hi.is (N.D.O.); gudny@hjarta.is (G.E.); johanna@hjarta.is (J.E.S.); v.gudnason@hjarta.is (V.G.); 3 Research Center of Movement Science, University of Iceland, Stapi v/Hringbraut, 101 Reykjavík, Iceland; thorasve@hi.is; 4 National Institute of Diabetes and Digestive and Kidney Diseases, Diabetes, Endocrinology, and Obesity Branch, Bethesda, MD 20892, USA; brychtar@niddk.nih.gov (R.J.B.); chenkong@niddk.nih.gov (K.Y.C.); 5 CAPHRI Care and Public Health Research Institute, Department of Social Medicine, Maastricht University, P.O. Box 616, 6200 MD Maastricht, The Netherlands; a.koster@maastrichtuniversity.nl; 6 Department of Biostatistics and Bioinformatics, Rollins School of Public Health, Emory University, Atlanta, GA 30322, USA; dvandom@emory.edu; 7 Department of Sports Science and Clinical Biomechanics, University of Southern Denmark, 5230 Odense M, Denmark; PCaserotti@health.sdu.dk; 8 Center for Sport and Health Sciences, University of Iceland, Saemundargata 2, 101 Reykjavik, Iceland; erljo@hi.is; 9 Department of Sport and Physical Activity, Western Norway University of Applied Science, Bergen 5063, Norway; 10 Laboratory of Epidemiology and Population Sciences, National Institute on Aging, Bethesda, MD 20892, USA; LaunerL@nia.nih.gov (L.J.L.); harris99@nia.nih.gov (T.B.H.); 11 Faculty of Medicine, School of Health Sciences, University of Iceland, Saemundargata 2, 101 Reykjavik, Iceland

**Keywords:** seasonal, aging, physical activity, accelerometer

## Abstract

In Iceland, there is a large variation in daylight between summer and winter. The aim of the study was to identify how this large variation influences physical activity (PA) and sedentary behavior (SB). Free living PA was measured by a waist-worn accelerometer for one week during waking hours in 138 community-dwelling older adults (61.1% women, 80.3 ± 4.9 years) during summer and winter months. In general, SB occupied about 75% of the registered wear-time and was highly correlated with age (β = 0.36). Although the differences were small, more time was spent during the summer in all PA categories, except for the moderate-to-vigorous PA (MVPA), and SB was reduced. More lifestyle PA (LSPA) was accumulated in ≥5-min bouts during summer than winter, especially among highly active participants. This information could be important for policy makers and health professionals working with older adults. Accounting for seasonal difference is necessary in analyzing SB and PA data.

## 1. Introduction

The benefits of physical activity (PA) are well known and important for disease prevention and the maintenance of self-support in older adults [1,2]. In 2008, the U.S. Department of Health and Human Services published guidelines for Americans that recommended older adults conduct at least 150 min × week^−1^ of moderate intensity PA or 75 min × week^−1^ of vigorous-intensity aerobic PA, or an equivalent combination of moderate and vigorous PA (MVPA) [3]. The guidelines state that aerobic PA should be performed in episodes of at least 10 min bouts, which has been supported by other researchers [4,5,6]. Current Icelandic PA guidelines also reflect these findings and suggest that older adults perform a minimum of 30 min of daily moderate activity, accumulated in 10–15 min bouts [7]. However, other studies have shown that health benefits might also be gained with bouts of activity that last less than 10 min [8,9] and by avoiding sitting and sedentary behavior (SB) [10,11]. In studies of free-living PA in older adults, accumulation of MVPA in bouts as low as five minutes has been reported [12].

Levels of PA tend to change according to season in younger adults [13,14,15,16,17,18,19,20]. In 2007, Tucker and Gilliland [21] reviewed 37 studies conducted between 1980 and 2006 and found that there were significant seasonal changes in MVPA in 73% of the studies. Reasons for seasonal differences in PA may include changes in daylight hours [22], outdoor temperature [22,23,24,25], precipitation, and wind [21,26,27] that occur throughout the year. In Iceland, cold temperatures, rain, snow, and wind are frequent events throughout the year, but are exacerbated in the winter. Daylight length changes dramatically with season in Iceland, with close to 24 h of daylight in summer and very little daylight in winter. The combination of prolonged darkness and harsh, slippery conditions in winter makes outdoor PA challenging, especially for older populations [21,22,23,24,25,27,28].

Various subject characteristics including age, sex, body-mass-index (BMI) status, and average PA level may play a role in seasonal PA variability [16]. Until now, most studies of seasonal changes in PA have been conducted in children, adolescents, or young adults [21], and many used self-reported PA, which may bias reports of PA and SB [29,30]. A few studies using objective measurements have shown older people to be less active during the winter [23,31,32]. A previous study using both questionnaires and objective PA measures in an adult cohort with a broad age range (20–70 years) observed limited seasonal variability in non-occupational PA for sedentary individuals compared to regular exercisers [16], but the influence of mean activity level on seasonal variability in older populations (>70 years) is unknown. It is conceivable that older adults who are more motivated to be physically active would take greater advantage of favorable summer weather and be more affected by adverse environmental conditions in winter than those with lower mean activity levels throughout the year.

The aim of this study was to compare objectively assessed free-living PA and SB patterns during summer and winter periods in a sample of older community-dwelling Icelanders from the Age, Gene/Environment Susceptibility Reykjavik Study (AGESII study). We measured two one-week assessment periods during the summer and winter months to investigate the seasonal influences on PA and SB patterns, including time spent in different PA intensity categories and in bouts. To our knowledge, this is the first study to compare seasonal changes in free-living objectively measured PA and SB in an older community-dwelling population, where the daylight change is dramatic between summer and winter months.

## 2. Materials and Methods

### 2.1. Study Population and Design

This study was a part of the AGESII study [33]. The current study focuses on data collected between April 2009 and June 2010, when objective PA measurement by accelerometers was added to the AGESII study protocol. During the PA measurement period, 1194 subjects participated in the AGESII study (the flow chart for this study population is shown in Figure S1). For the PA measurements, participants (*n* = 55) were excluded due to cognitive impairment (MMSE < 20), as those participants were not expected to be able to reliably wear and use the accelerometer [34], 95 were excluded for other reasons (e.g., blindness and other physical obstructions), 84 refused and 294 did not participate because of scheduling conflicts. Five subjects lost the accelerometers and 12 files were unusable because of device failures. The remaining 649 (54.4%) participants had accelerometry data. Of these, there were 590 participants who had four or more valid days (≥10 h of wear time) of useable accelerometry data. Earlier, it has been shown that participants who wore the accelerometers had similar characteristics compared with those participants who did not receive an accelerometer [35]. These 590 subjects were used for the cross-sectional analysis of the influence of day length and temperature on PA and SB patterns (Table S1).

To improve the sensitivity of detecting differences between seasons, we carried out a within-individual nested study We asked two hundred and nineteen (219) subjects who had worn an accelerometer during the “summer” months with more hours of daylight (from 15 May to 30 September 2009) to wear the monitor again for a week during the “winter” months with fewer daylight hours (from 18 November 2009 to 19 March 2010). 160 subjects accepted participation, and three device malfunctions led to 157 participants with usable accelerometer data, 138 of which had four or more valid days of measurements in both winter and summer sessions and were used for the final primary analysis. The summer-winter sub-group was found to reflect the total participants measured in the original accelerometry study. No measurements were done in July 2009 because of summer vacations and in late December 2009 because of Christmas. The study was approved by the Icelandic National Bioethics Committee (VSN: 00-063), the Icelandic Data Protection Authority, and the institutional review board of the U.S. National Institute on Aging, National Institutes of Health. Signed informed consent was given by all participants.

### 2.2. Demographic and Environmental Parameters

Participants came to the Icelandic Heart Association in Kópavogur, Iceland, for assessment of cognitive and physical function as part of the AGESII study. Height and weight were taken using standardized procedures and body mass index (BMI) was computed as the ratio of weight and height^2^ (kg × m^−2^). Participants reported overall health status on a discrete scale (1-excellent, 2-very good, 3-good, 4-fair, 5-poor) and gender. The hours of daylight of each participant’s week of free-living measurement were obtained using the Sunrise/Sunset calculator provided by the Earth System Research Laboratory of the National Oceanic and Atmospheric Administration (http://www.esrl.noaa.gov/gmd/grad/solcalc/calcdetails.html). The average daily outdoor temperature over the same period was obtained from the Weather Underground (www.wunderground.com) historical weather data.

### 2.3. Assessment of PA

Participants wore the ActiGraph GT3X accelerometer (Actigraph Inc., Pensacola, FL, USA) at the right hip for one complete week and were instructed to remove the monitor only during sleep, showers, bathing, or other water activities. Non-wear was defined as a period of at least 60 consecutive minutes during which the activity monitor recorded zero counts in all axes, allowing 1–2 min of vertical-axis counts between 0 and 100. A day of accelerometer wear was considered valid if the wear time was ≥10 h. Participants with fewer than four valid days over the week of measurement were excluded from further analysis [36]. To explore the general patterns of PA, we only report the data in the vertical axis and present daily averages of total PA (TPA) (counts × day^−1^). Activity intensity categories were defined as: sedentary behavior (SB) < 100 count × min^−1^ during wear time, low-light PA (LIPA) as 100–759 counts × min^−1^, lifestyle PA (LSPA) ≥ 760 counts × min^−1^ and MVPA ≥ 2020 counts × min^−1^ [35,37,38]. A bout of LSPA was defined as at least five consecutive minutes of activity counts above the LSPA threshold (≥760 counts × min^−1^), allowing for one minute outside of the threshold [36,38]. We focused on LIPA and LSPA, as well as a lower bout window in LSPA due to the age of our study participants [12]. All PA variables and the SB variables were extracted using customized software programmed in Matlab version R2013a (The Mathworks, Inc., Natick, MA, USA).

### 2.4. Statistical Analyzes

SAS 9.4 (SAS Institute Inc., Cary, NC, USA) was used for statistical analysis. For the primary analysis (two one-week free-living measurements), an ANCOVA adjusted for repeated measures (SAS mixed model procedures) was used to explore the association between the accelerometer variables and temperature and day length, while adjusting for age, sex, BMI, and health status. To adjust for the skewness, PA variables were square root transformed and all parametric statistical tests were conducted on transformed data (all average numbers in texts, tables and graphs were produced from original not transformed data). The standardized β values were used to compare the relative strength of contributions by each variable to PA. For additional analyses, the following procedures were implemented. Due to high frequency of zeros, paired, nonparametric comparisons (Wilcoxon Signed Rank test) were used to test for seasonal difference in the number of bouts, accumulated minutes of bouted activity, and total counts accumulated in ≥5-min bouts of LSPA. In our data, only eight subjects had 10-min bouts of MVPA (six in the winter, two in the summer, in which none had in both seasons), and 24 subjects had PA in 10-min bouts of LSPA (15 in the winter, 18 in the summer, in which nine had in both seasons). To determine whether seasonal effects differed by participant activity levels (interaction between activity level and seasons), the group was separated by the median of TPA (averaging summer and winter) to high activity and low activity. The Wilcoxon test was also used to compare the accumulated minutes and counts between low- and high activity participants. The Wilcoxon test was used to compare summer to winter change between groups (sex and low vs. high activity participants; group-season interaction). Results of the bouted activity are presented as lower quartile (LQ), median value (MD), and upper quartile (UQ). Low- and high-activity participants were separated by the median of the mean TPA for summer and winter. McNemar’s test was then used to compare summer and winter proportions of participants who had at least one bout of ≥5-min LSPA. Chi-Square test was used to compare groups.

## 3. Results

### 3.1. Demographic and Environmental Measures

Descriptive statistics for the 138 individuals who had complete valid measurements during both summer and winter are displayed in Table 1. The mean age of the participants was 80.3 years with a range between 73 to 91 years (60.1% women). The average self-reported health status was good to very good (2.6 ± 1.2). As expected, participants experienced more daylight and higher temperatures during the summer compared to during the winter. All PA variables were significantly higher (*p*’s < 0.05) except for MVPA, while significantly less time was spent in SB (*p* < 0.05) in the summer. Only nine individuals had mean MVPA greater or equal to 30 min per day (eight in the winter, four in the summer, including three in both seasons). Moreover, 19 individuals had mean MVPA greater or equal to 20 min per day (13 each in winter and summer, with seven in both seasons). These subjects were younger (*p* = 0.003) and tended to have lower BMI (*p* = 0.11) and report better health status (*p* = 0.07) than the rest of the group.

### 3.2. Predictors of Physical Activity

Results of the mixed model ANCOVA are shown in Table 2. We tested separate models for each of the seasonal variables (day length and temperature) due to high collinearity. Age had the most significant negative associations with all PA variables, with the standard β values from −0.32 to −0.44, and a positive association with SB (β = 0.36). There was an inverse association between BMI and all PA variables, with β values from −0.16 to −0.24, and a positive association was between SB and BMI (β = 0.18). Women had more LIPA (β = 0.16) compared to men. Self-reported health status was not associated with any of the PA variables. Adjusting the PA for differences in wear-time had little impact on the standardized β values or confidence intervals (CI). Cross-sectional regression analysis performed on the one week PA and SB data from the cross-sectional sample (590 subjects) showed similar patterns and trends as our observations in the primary analysis using within-individual data in summer vs. winter (shown in Table S1).

### 3.3. Bouts of Physical Activity

To further explore if the changes in PA between seasons occurred into bouts, participants were classified in to two groups, high activity and low activity. Most of the high activity participants achieved at least one ≥5-min bout of LSPA both during summer and winter, but only 58% the low activity participants achieved at least one ≥5-min bout of LSPA during the winter and around 78% during the summer (Table 3). The number of bouts, counts and minutes accumulated in ≥5-min bouts of LSPA were higher during the summer compared with winter in both low- and high-activity participants. Moreover, the differences between summer and winter were significantly higher for high activity participants than for low activity participants (Table 3).

## 4. Discussion

This study focused on seasonal changes in free-living objectively measured PA and SB in older community-dwelling Icelanders, where there is considerable daylight variability throughout the year. The main findings of this study are that both men and women were more active in summer compared to winter, with more PA (about 19%) and less time spent in SB (4.4% in men, 2.5% in women). Furthermore, results revealed that age was the strongest predictor of PA and more SB. Participants accumulated more LSPA in bouts during the summer compared to winter and this difference was greater in high activity participants than low activity participants.

Our findings were similar to some previous studies in young to middle-aged adults in the UK [20], in the U.S. [13], and in older adults in the UK [31] and Japan [23], where PA during winters was 10–20% less than other seasons. Step count in UK adults decreased during winters [14,20], and the same was shown in a study of U.S. adults [15]. Self-reported data has also shown a decrease in PA during winters in UK adults [39], U.S. adults [16], and in Canadian adults [40].

On average, older community-dwelling Icelandic men and women spent 80% of their PA time in the LIPA intensity category, both during summer and winter, followed by LSPA intensity, which was only 37 min per day during the summer and about 29 min per day during winter. Only a small percentage (14%) of our study participants engaged in MVPA for more than 20 min per day. Women only achieved 4–5 min of MVPA per day on average. LIPA tends to be accumulated by incidental activities, like shopping and walking at low pace [20], while LSPA is accumulated by more structured activities like walking, vacuuming and cleaning [38,41]. This distribution of activity is similar to previous accelerometry-based studies in older adults [31] and younger women [13] where less time was spent in light intensity PA during the winter.

A large proportion of wear time, ~75% or over 10 h per day, was spent sedentary. Similar sedentary time was observed in a previous study of older adults with comparable mean age (78 years) [31] and in slightly older study populations (≥80 years) [42,43]. Although SB was statistically lower in the summer compared to winter, the actual change was quite small: 14 min for men and 6 min for women. Seasonal changes in SB have been observed in younger adults [13,20] as well as in middle-aged and older U.S. adults [44] where individuals were more likely to exhibit patterns of prolonged SB during the winter.

Length of daylight has been shown to have influence on the total amount of PA, but it varies between seasons and countries [16,22,26,27,28,39,45]. In our study, the average difference in daylight between seasonal visits was around 7.5 h. The change in temperature between seasons was not as dramatic, only around 6 °C in mean temperature. If we compare these ranges to the ranges observed in a Japanese study [27], the variation in temperature in Iceland is much smaller, while the daylight length had a much wider range. On days with higher temperature or longer daylight, almost all types of PA were higher. Conversely, lower temperature and shorter daylight were associated with more SB. Although the daylight difference is dramatic, the relative difference in PA (2.8–17.2%) and SB (0.4–1.2%) are relatively small in comparison. It might be speculated that the population is well adapted to the changes in daylight.

The strength of the study is that we investigated seasonal changes in an older population living at a high latitude, where the daylight change is dramatic between summer and winter months. Findings are based on the well-characterized large-population-based cohort of older Icelandic adults. The participation in the study was excellent, with 73.1% (*n* = 160) of subjects agreeing to participate, and the compliance was high, as 88.6% of the participants had four valid days of measurements. The use of objective measurements is thought to measure changes in free-living PA and SB more accurately than questionnaires [29,30]. There are also some limitations that need to be accounted for when interpreting the results. Accelerometers miss some movement patterns, like upper body movements during activities like heavy load carrying and lifting, although such events are less likely in our older population. They are also limited in detecting non-ambulatory activities like cycling [46] and water activities like swimming [47]. Swimming is a quite popular exercise form in Iceland, and about a quarter of all participants reported swimming as an exercise both during summer and winter. But for those who reported swimming, only 25% swam for >30 min each time [35]. The influence of daylight and temperature could not be separated in this study.

## 5. Conclusions

Our data showed that older community-dwelling Icelandic men and women were more physically active and less sedentary during the summer as compared to the winter; however, the differences in the variables were relatively small. High activity participants experienced more changes in LSPA bouts from summer to winter compared to low activity participants. Thus, in studies on PA and SB in older people, it is important to consider seasonal differences. These observations may also be useful for policy makers and health professionals working to increase PA and/or reduce SB in older adults.

## Figures and Tables

**Table 1:** Demographic, environmental and activity parameters (mean and standard deviation (SD)) for participants with complete data during summer and winter.

**Demographics**						
N	138 (F = 83; M = 55)					
Age, year	80.3 (4.9)					
Height, cm	167.4 (9.1)					
Weight, kg	75.8 (14.5)					
BMI, kg × m^−2^	27.0 (4.5)					
Self-Reported Health (1-excellent, 5-poor)	2.6 (1.2)					
	**Summer**			**Winter**		
	**Men**	**Women**	**All**	**Men**	**Women**	**All**
**Environmental**						
Average Day Length (h:min × day ^−1^)	15:26 (2:51)	14:41 (2:38)	14:59 (2:45)	7:26 (2:04)	7:16 (1:55)	7:20 (1:58)
Average Outdoor Temperature, °C	8.4 (2.3)	8.6 (2.5)	8.5 (2.4)	2.7 (2.5)	2.5 (2.7)	2.6 (2.6)
**PA Parameters**						
Wear time (min × day^−1^)	13:56 (1:19)	13:39 (1:14)	13:46 (1:16)	13:35 (1:15)	13:25 (1:07)	13:29 (1:10)
TPA (×1000 counts × day^−1^)	118 (83)	106 (60)	111 (68)	99 (66)	89 (46)	93 (55)
LIPA (h:min × day^−1^)	2:48 (1:02)	2:58 (1:02)	2:54 (1:02)	2:21 (0:52)	2:48 (1:01)	2:38 (0:59)
LSPA (h:min × day^−1^)	0:40 (0:37)	0:34 (0:28)	0:36 (0:32)	0:31 (0:27)	0:24 (0:18)	0:27 (0:22)
MVPA (h:min × day^−1^)	0:09 (0:16)	0:05 (0:06)	0:07 (0:11)	0:09 (0:13)	0:04 (0:07)	0:06 (0:10)
SB (h:min × day^−1^)	10:29 (1:25)	10:07 (1:16)	10:15 (1:20)	10:43 (1:21)	10:13 (1:18)	10:25 (1:20)
WT-SB (percent of wear time)	75.2 (10.0)	74.1 (9.3)	74.5 (9.7)	78.9 (9.9)	76.1 (9.7)	77.2 (9.9)

F = female; M = male; PA = Physical activity; SB = Sedentary behavior; TPA = Total PA; WT-SB = SB variable normalized for wear time; LIPA = Low-intensity PA (100–759 counts × min^−1^); LSPA = Lifestyle PA (≥760 counts × min^−1^); MVPA = Moderate- to-vigorous PA (≥2020 counts × min^−1^).

**Table 2:** Results of the ANCOVA for of PA and SB parameters for the 138 participants with complete data during summer and winter. Covariates included age, sex, BMI, self-reported health status, day length, and temperature. Data are presented as standardized β (β), 95% confidence interval (95% CI). None of the included covariates affected the standardized as for the two seasonal variables, Temperature and Day length. A negative β value indicates an inverse relationship. Significance (*p* < 0.05) are shown in bold. Two separate models were run for each variable, where only one seasonal variable (temperature or day length) was in each model; seasonal variables with higher β are marked with **^†^**. Other variables were included in both models but are shown in the table only for the model with the higher β of the seasonal variables.

Variables		Temperature/Day Length		Age		Female		BMI		Health Status	
		β	95% CI	β	95% CI	β	95% CI	β	95% CI	β	95% CI
WT-SB	Temperature	**−0.16 ^†^**	**−0.21; −0.10 ^†^**	**0.36**	**0.21; 0.50**	−0.12	−0.27; 0.02	**0.18**	**0.03; 0.33**	0.07	−0.08; 0.22
	Day length	**−0.16**	**−0.21; −0.11**								
TPA *^a^*	Temperature	**0.17 ^†^**	**0.11; 0.22 ^†^**	**−0.44**	**−0.58; −0.29**	−0.04	−0.18; 0.10	**−0.21**	**−0.35**; **−0.06**	−0.11	−0.25; −0.03
	Day length	**0.14**	**0.09; 0.19**								
LIPA *^a^*	Temperature	**0.14**	**0.08; 0.19**								
	Day length	**0.15 ^†^**	**0.10; 0.21 ^†^**	**−0.32**	**−0.47; −0.17**	**0.16**	**0.01; 0.30**	**−0.16**	**−0.31; −0.01**	−0.01	−0.17; 0.14
LSPA *^a^*	Temperature	**0.18 ^†^**	**0.12; 0.24 ^†^**	**−0.43**	**−0.57; −0.29**	−0.06	−0.20; 0.08	**−0.17**	**−0.31; −0.02**	−0.13	−0.27; −0.01
	Day length	**0.15**	**0.10; 0.21**								
MVPA *^a^*	Temperature	**0.08 ^†^**	**0.01; 0.14 ^†^**	**−0.33**	**−0.47; −0.19**	−0.13	−0.27; 0.01	**−0.24**	**−0.39; −0.10**	−0.11	−0.26; 0.03
	Day length	0.05	−0.02; 0.11								

PA = Physical activity; SB = Sedentary behavior; WT-SB = SB variable normalized for wear time; TPA = Total PA; LIPA = Low-intensity PA (100–759 counts × min^−1^); LSPA = Lifestyle PA (≥760 counts × min^−1^); MVPA = Moderate-to-vigorous PA (≥2020 counts × min^−1^); *a* = Square root transformed; **^†^** = seasonal variable with higher β.

**Table 3:** Median value (MD) and inter quartile limits (IQL) for the means of the valid day values, for ≥5-min bouts of LSPA for sub-population of participants with repeat visits during summer and winter, presented separately for low- and high-active participants. Low vs. high active participants were separated by the median of average TPA for summer and winter. Also, the proportion of participants who reached any bout of ≥5-min of LSPA.

	Low Active (*n* = 69)		High Active (*n* = 69)		*p*	*p*	*p*
	Winter	Summer	Winter	Summer	Season	Low/High	Interaction
Number of participants who achieved at least one bout of LSPA ≥ 5-min	40 (58.0%)	54 (78.4%)	69 (100%)	68 (98.6%)	**0.012 ^a^**	**<0.0001 ^b^**	**0.002 ^c^**
All subjects with repeated measures during summer and winter	MD (IQL)	MD (IQL)	MD (IQL)	MD (IQL)			
Number of ≥5-min bouts of LSPA (day^−1^)	0.17 (0; 0.50)	0.33 (0.14; 0.86)	1.5 (1.0; 2.43)	2.4 (1.29; 3.71)	**<0.0001 ^d^**	**<0.0001 ^e^**	**0.001 ^c^**
Counts accumulated in ≥5-min bouts of LSPA (counts × day^−1^)	1033 (0; 4238)	3600 (867; 8136)	18,608 (9135; 55,740)	31,952 (17,574; 70,021)	**0.0005 ^d^**	**<0.0001 ^e^**	**0.035 ^c^**
Minutes accumulated in ≥5-min bouts of LSPA (min:s × day^−1^)	0:50 (0; 3:26)	2:40 (0:50; 5:43)	12:43 (7:10; 26:08)	22:17 (10:06; 35:40)	**<0.0001 ^d^**	**<0.0001 ^e^**	**0.008 ^c^**

LSPA = Lifestyle PA (≥760 counts × min^−1^); MD = Median value; IQL = Inter quartile limits; ^a^ = McNemar’s test used to compare summer and winter; ^b^ = Chi-Square test, low- and high active compared; ^c^ = Wilcoxon test of summer to winter change between low vs. high active participants (season-TPA interaction); ^d^ = Wilcoxon Singed Rank test used to compare winter and summer; ^e^ = Wilcoxon test, low- and high active compared. Significant relationship is bolded.

## Supplementary Materials

The following are available online at www.mdpi.com/1660-4601/14/10/1268/s1. Figure S1: Flow chart for the study population, Table S1: Results of backward-elimination, linear regression of cross-sectional PA and SB parameters for the AGESII cohort. Covariates included age, sex, BMI, self-reported health status, day length, and temperature. Data are presented as standardized Beta. A negative standardized Beta (β) value indicates an inverse relationship.
