# Supplementary material for: Comparison of Summer and Winter Objectively Measured Physical Activity and Sedentary Behavior in Older Adults: Age, Gene/Environment Susceptibility Reykjavik Study
Source: Int J Environ Res Public Health. 2017 Oct 21;14(10):1268. doi: 10.3390/ijerph14101268 (PMC5664769; doi:10.3390/ijerph14101268)
Supplement: Supplementary file 1 [file ijerph-14-01268-s001.pdf]

Figure S1. Flow chart for the study population.

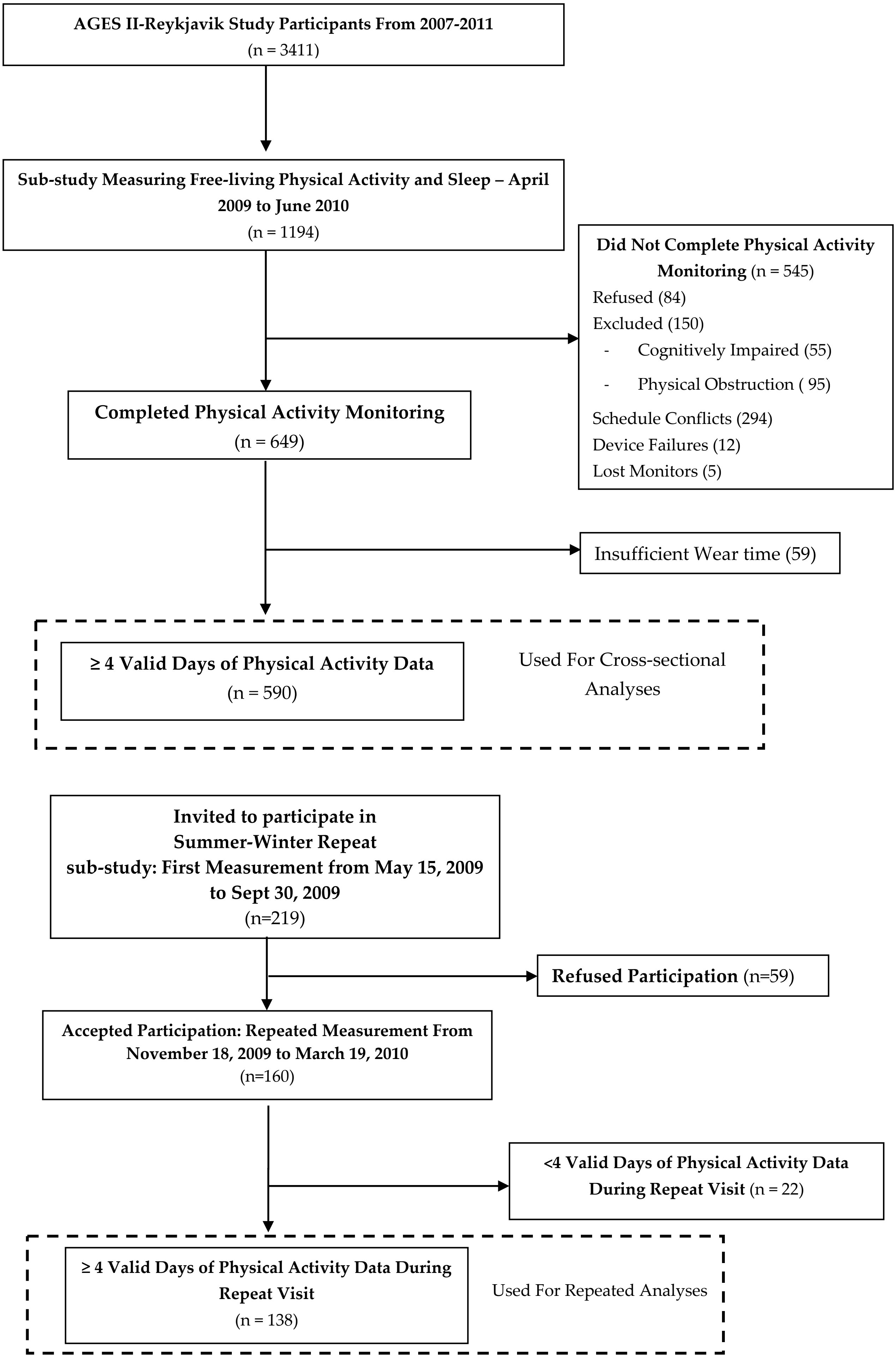

Table S1. Results of backward-elimination, linear regression of cross-sectional PA and SB parameters for the AGESII cohort. Covariates included age, sex, BMI, self-reported health status, day length, and temperature. Data are presented as standardized Beta. A negative standardized Beta (β) value indicates an inverse relationship.

Variables		Temperature/Day length		Age		Female		BMI		Health status	
		β	95%CI	β	95%CI	β	95%CI	β	95%CI	β	95%CI
WT-SB[a]	Temperature	**−0.10**	**−0.17: −0.02**								
	Day length			**0.31**	**0.23; 0.38**	**−0.15**	**−0.23; −0.08**	**0.11**	**0.03; 0.19**	**0.10**	**0.01; 0.17**
TPA[b]	Temperature	**0.10**	**0.03; 0.17**	**−0.37**	**−0.45; −0.30**	−0.04	−0.11; 0.04				
	Day length							**−0.14**	**−0.22; −0.06**	**−0.11**	**−0.18; −0.03**
LIPA[b]	Temperature										
	Day length	0.06	−0.02; 0.13	**−0.29**	**−0.36; −0.21**	0.19	0.11; 0.26	**−0.09**	**−0.17; −0.01**	**−0.09**	**−0.16; −0.002**
LSPA[b]	Temperature	**0.11**	**0.038; 0.19**	**−0.36**	**−0.43; −0.28**	**−0.08**	**−0.16; −0.006**				
	Day length							**−0.11**	**−0.19; −0.03**	**−0.12**	**−0.19; −0.04**
MVPA[b]	Temperature	0.05	−0.02; 0.13	**−0.29**	**−0.37; −0.21**	**−0.21**	**−0.29; −0.14**				
	Day length							**−0.18**	**−0.25; −0.10**	**−0.10**	**−0.17; −0.02**

WT= Wear time; SD= standard deviation, day to day variation in each PA/SB variable; PA= Physical activity; SB= Sedentary behavior; TPA= Total PA; LIPA= Low-intensity PA (100-759 counts × min^{-1}); LSPA= Lifestyle PA (≥760 counts × min^{-1}); MVPA= Moderate-to-vigorous PA (≥2020 counts × min^{-1}); *a*= adjusted for wear time; *b*= square root transformed.
